# Assessing the unconventional reservoirs of the Nukhul formation in the Rudeis-Sidri Field, Gulf of Suez: petrophysical characterization and flow unit discrimination

**DOI:** 10.1038/s41598-026-49085-y

**Published:** 2026-05-11

**Authors:** Marwa Z. El-Sawy, Bassem S. Nabawy, Tarek F. Shazly, Ahmed H. Saleh

**Affiliations:** 1 Exploration Department, Egyptian Petroleum research Institute (EPRI), Cairo, Egypt; 2 Department of Geophysical Sciences, National Research Centre, Giza, Egypt; 3 Geology Department, Faculty of science, Zagazig University, Zagazig, 44519 Egypt

**Keywords:** Unconventional reservoirs, Petrophysical analysis, Reservoir heterogeneity, Hydraulic flow units (HFU), Hydrocarbon potential, Energy science and technology, Engineering, Solid Earth sciences

## Abstract

This study employed an integrated petrophysical workflow that combines conventional well-log interpretation with routine core analysis to evaluate the Nukhul Formation in the Rudeis–Sidri Field and to assess its hydrocarbon potential quantitatively. The Sidri-14 well, Gulf of Suez, penetrates the Lower Miocene Nukhul Formation, a sequence of sandstone, shale, and limestone. Petrophysical evaluation, supported by core (215 samples) and well log analysis, subdivided the formation into four reservoir units (A–D). Units A, B, and C exhibit low porosity (4.3–13.6%), and low permeability (0.03–13.099 mD), with a heterogeneity coefficient (V) of 0.92, classifying the formation as highly heterogeneous with variable but moderate hydrocarbon saturation, while water saturation (Sw) frequently exceeds 50%, limiting net pay, whereas Unit D, being carbonate-dominated, shows no reservoir potential. The porosity–permeability relationship (R^2^ = 0.93–0.98) confirms that permeability is strongly controlled by pore-throat size. By employing hydraulic flow unit (HFU) classification (via RQI, NPI, FZI, and stratigraphic Lorenz techniques), most samples fall within tight/poor quality domains (RQI < 0.25 μm, R_35_ < 1 μm), requiring stimulation for economic recovery. Hydraulic Flow Unit (HFU) classification subdivides the reservoir into 8 HFUs, where HFU-1 and HFU-2 contribute the largest portion of flow (14.11% and 16.52% of total flow capacity) and equivalent to PSRT1 and PSRT2**,** which are characterized by the highest RQI and FZI values, wide pore-throat sizes, high permeability, and excellent flow capacity, while HFU-8 contributes 0%, highlighting uneven productivity distribution. This integration reveals that the Nukhul Formation is classified as an unconventional, tight reservoir with limited but heterogeneous hydrocarbon potential. Productive intervals exist but are restricted to specific HFUs, meaning advanced recovery techniques (hydraulic fracturing, horizontal drilling) are essential for efficient hydrocarbon exploitation.

## Introduction

**Fig. 1:**
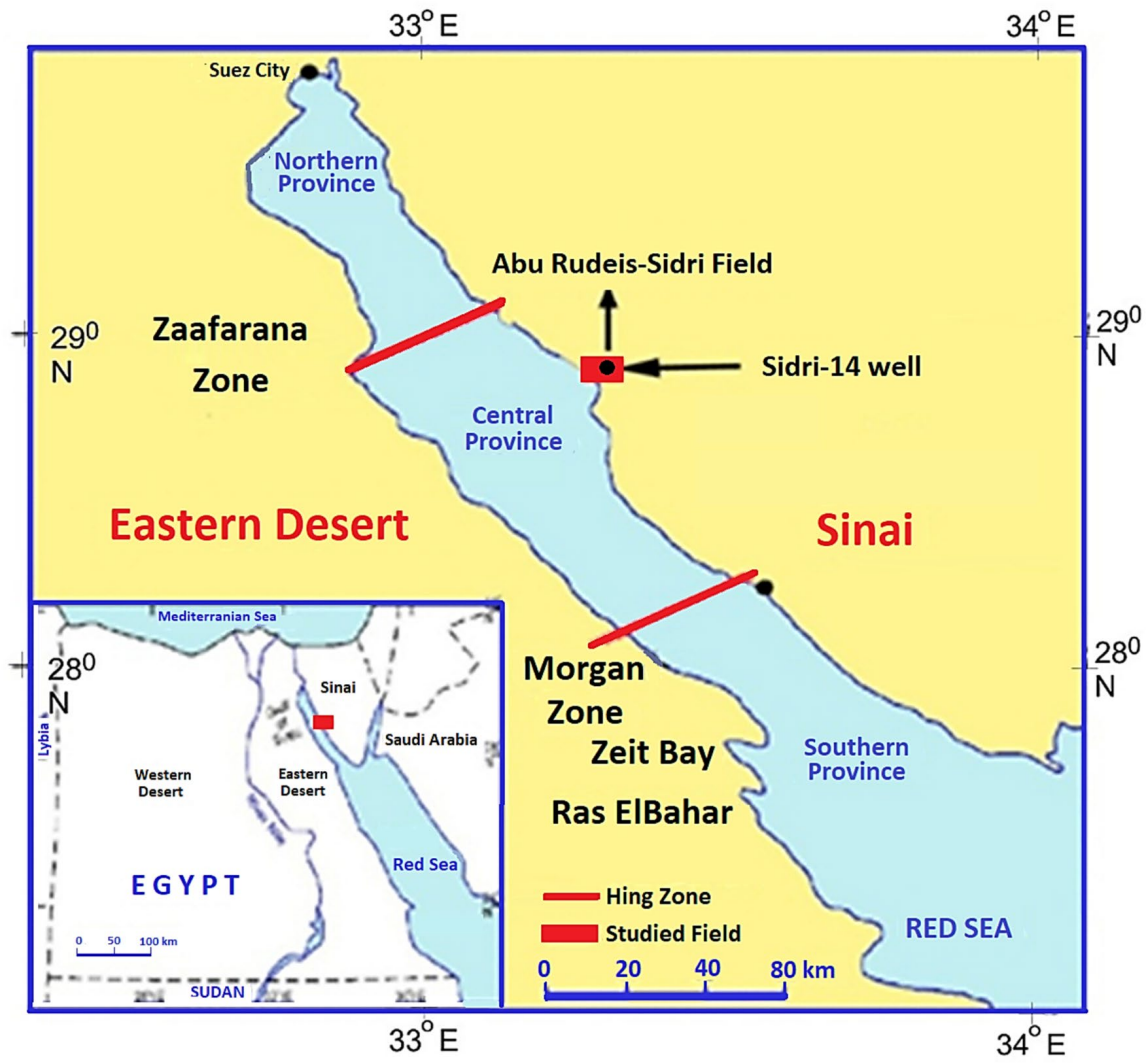
Location Map of the studied Rudeis-Sidri Field showing the main structural provinces of the Gulf of the Suez (modified after EGPC, 1996)^3^.

The Red Sea–Gulf of Suez rift system contains numerous sedimentary basins that hold abundant hydrocarbon resources. Within this system, the study area lies in the north-central Gulf of Suez, a region that has become the focus of intensive geological and petroleum research due to its structural complexity and proven reserves. The Rudeis–Sidri Field, which is more than 89 km^2^ in size, is one of the most prolific oil-producing fields in this part of the rift^1^. Geographically, it is positioned southeast of the October and Ras Budran fields and approximately 25 km north of the Belayim Land field (Fig. 1). The field occupies a structurally favorable setting along major fault blocks, which have acted both as hydrocarbon traps and as pathways for fluid migration^2^. This unique position makes the Rudeis–Sidri Field a key contributor to Gulf of Suez petroleum systems and an ideal site for detailed reservoir characterization studies such as those conducted on the Sidri-14 well.

Exploration in the Rudeis-Sidri Field commenced in March 1947 with the drilling of the Rudeis-01 well, which was unsuccessful and reported as dry. However, the subsequent drilling of the Rudeis-02 well represents a significant milestone in the field, as it yielded the first productive discovery of hydrocarbons within the field. This discovery highlighted the reservoir potential of the syn-rift Miocene sandstones and guided further exploration. In the following years, numerous productive wells were drilled, not only to appraise the Nukhul Formation but also to investigate the hydrocarbon potential of the underlying pre-Miocene reservoirs^3–5^. Today, the Rudeis-Sidri Field produces an average of 2700 barrels of oil per day (BOPD), primarily from the Nukhul Formation, with secondary contributions from the Thebes Limestone, Matulla Formation, Turonian carbonates, and Nubia Sandstone. This production profile underscores the multiphase petroleum system of the field while reaffirming the Nukhul as the principal reservoir unit. The long production history also provides valuable data for modern reservoir characterization, including well log interpretation, core analysis, and hydraulic flow unit modeling, as carried out in the Sidri-14 well^6^.

Petrophysical analysis is widely recognized as one of the most effective methods for reservoir characterization and the classification of hydraulic flow units (HFUs), as it integrates geological, petrophysical, and engineering data into a unified evaluation framework^7–10^. Recent studies have shown the value of combining core plug measurements with well log data to improve the accuracy of porosity–permeability relationships, flow zone indicator (FZI) determination, and reservoir quality prediction^11–14^.

Accordingly, the primary objective of this study is to assess the hydrocarbon potential of the Lower Miocene reservoirs, with a particular focus on the Nukhul Formation in the Sidri-14 well. This has been achieved by integrating routine core analysis with the interpretation of conventional well logging tools (gamma ray, density, neutron, sonic, and resistivity logs). Such an integrated approach provides a reliable calibration for the petrophysical parameters, enabling the determination of shale volume, effective porosity, water saturation, hydrocarbon saturation, and ultimately, the subdivision of the reservoir into distinct HFUs by using Techlog software. This methodology not only refines the reservoir quality evaluation but also enhances the prediction of productive zones and hydrocarbon distribution within the structurally complex Gulf of Suez Basin^15–18^.

By applying the hydraulic flow unit (HFU) concept of^11^ to the available core intervals, reservoir layers exhibiting similar petrophysical characteristics were grouped into discrete flow units. This methodology allows for the integration of porosity–permeability relationships with reservoir quality parameters, ensuring a more reliable classification of flow behavior. Following this approach, the Nukhul reservoir in the Sidri-14 well was subdivided into HFUs by combining intervals with comparable rock quality indicators^19–22^. Each HFU is characterized by a distinct range of values for the Flow Zone Indicator (FZI) and Reservoir Quality Index (RQI), which serve as diagnostic parameters for differentiating reservoir quality and productivity.

Moreover, various mathematical relationships have been developed and substantiated in prior research to directly predict reservoir quality characteristics from core and well-log data. This encompasses equations for the Reservoir Quality Index (RQI), Normalized Porosity Index (NPI), Flow Zone Indicator (FZI), and the Reservoir Potentiality Index (RPI)^20^. The utilization of these models offers a quantitative framework for assessing reservoir heterogeneity, pinpointing productive intervals, and improving the prediction of hydrocarbon deliverability within the Nukhul Formation.

## Geologic setting

Between the Late Oligocene (~28 Ma) and the end of the Miocene (~5.33 Ma), the Gulf of Suez Rift was an active continental rift basin formed in response to the extensional tectonics that also gave rise to the Red Sea Rift. It trends NW–SE for about 300 km, with a width of 60–80 km, and forms the tectonic boundary between the Sinai Peninsula to the east and the Eastern Desert of Egypt to the west^23^. Until the Middle Miocene, the Gulf of Suez acted as a direct northwestern continuation of the Red Sea Rift system, after which both basins evolved independently due to changes in regional stress regimes and plate reorganization. This rifting episode strongly influenced sediment supply, accommodation space, and the development of a thick syn-rift stratigraphic succession. The structural framework is characterized by a series of half-grabens formed along large normal faults, producing significant accommodation space for thick sedimentary successions^24^.

The Gulf of Suez can be divided into three lithostratigraphic mega-sequences^25,26^. The first unit, (i) named a pre-rift, includes rocks that are the oldest sedimentary sequence from Cambrian to Eocene, providing excellent reservoirs and source rocks^27,28^, while the second one, (ii) syn-rift ranges from Oligocene to Miocene and provides hydrocarbon reservoir rocks (Nukhul, Rudeis, Kareem, and Belayim formations) and seal rocks (South Gharib, Zeit, and post-Zeit)^26,29,30^, and finally, the third recent rock unit, and (iii) from Pliocene (post-Miocene) to Holocene falls under post-rift^25,31–33^. The third one varies in thickness, lithology, areal distribution, depositional environments, and hydrocarbon importance^34–37^, and the study area is located and composed mainly of sandstones and interbedded with clays. The Gulf of Suez Rift and the studied area’s lithostratigraphy are comparable. The Nukhul Formation is a representation of the Early Miocene Aquitanian.

The Gulf is structurally segmented into three main depocenters: the northern (Abu Zenima-Zeit Bay), central (Belayim-October), and southern (Morgan-Ras Gharib) sectors, separated by accommodation zones and transfer faults. The tilted fault blocks form horst and graben systems that exert strong controls on sediment thickness and facies distribution. The basin architecture created favorable conditions for hydrocarbon entrapment, with tilted fault blocks and rollover anticlines forming the principal structural traps^38^.

The Gulf of Suez is Egypt’s oldest and most productive oil province. Its petroleum system relies on prolific Upper Cretaceous–Eocene source rocks, syn-rift sandstone and carbonate reservoirs, and multiple seal lithologies, including shales and evaporites. Hydrocarbon migration is strongly influenced by fault systems, with accumulations concentrated in structural highs such as the Belayim, Morgan, and October fields^25,39^.

Overall, the geologic setting of the Nukhul Formation in the Sidri-14 well highlights the influence of syn-rift tectonics and variable depositional environments on reservoir distribution and quality, making it a key target for hydrocarbon exploration and development in the Rudeis-Sidri Field.

## Lithostratigraphy and depositional environment

**Fig. 2:**
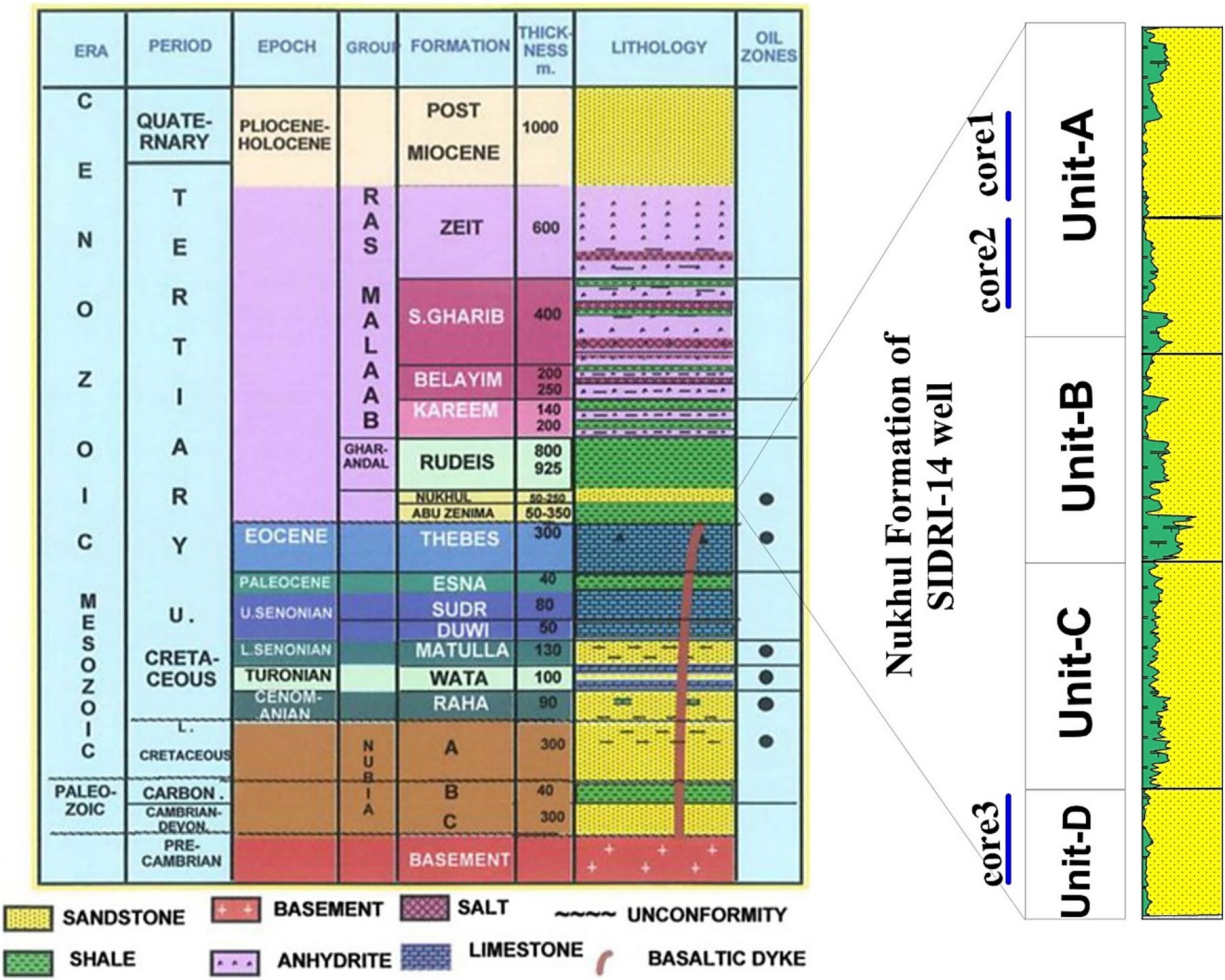
Schematic stratigraphic column of the studied formation showing the distribution of four units, cored interval, and the lithology.

The Rudeis–Sidri Oil Field, located in the central part of the Gulf of Suez Basin, is characterized by a well-developed syn-rift stratigraphic succession that reflects the tectonic and sedimentary evolution of the basin. The stratigraphy extends from Precambrian basement rocks through a thick sequence of Paleozoic to Cenozoic clastic and carbonate rocks, culminating in Quaternary-Holocene cover deposits (Fig. 2). The sandstone bodies act as hydrocarbon-bearing intervals, while the shale beds function as intraformational seals that enhance vertical heterogeneity^18^.

This succession records multiple depositional environments, ranging from deep marine to marginal marine and continental settings (marine transgressions and regressive episodes associated with the Oligocene–Miocene rifting). Within this stratigraphic framework, several formations play critical roles in the petroleum system^40^.

The Nukhul Formation (Early Miocene in age) is particularly important as a reservoir unit. It consists primarily of medium- to fine-grained sandstones interbedded with thin shale beds, deposited under shallow marine to marginal marine conditions during active rifting^41^. In the Sidri-14 well, detailed well log analysis and petrophysical evaluation enabled the subdivision of the Nukhul Formation into four reservoir units (A, B, C, and D) (Fig. 2) based on lithologic and petrophysical characteristics. The upper units (A and B) are typically cleaner and more porous, acting as the main oil-bearing intervals, whereas the lower units (C and D) are characterized by increased shale content and reduced reservoir quality. This subdivision highlights vertical variability in shale content, porosity, and hydrocarbon saturation, allowing for the identification of both pay and non-pay intervals. Such zonation is essential for building accurate reservoir models, assessing flow capacity, and guiding field development strategies.

Overall, the lithostratigraphy and depositional environment of the Nukhul Formation in the Sidri-14 well highlight the interplay between syn-rift tectonics, sediment supply, and marine incursions, which collectively shaped the vertical and lateral reservoir heterogeneity observed in the Rudeis-Sidri Oil Field^42^.

## Methodology

The studied well, drilled in the Rudeis–Sidri Oil Field, exhibits lithostratigraphic successions that are broadly similar from the seabed down to the Middle Miocene reservoir targets, with only minor variations in formation thickness. This study integrates multiple datasets, including well log interpretations and petrophysical core analyses, to provide a comprehensive characterization of the Nukhul Formation in the study area. By combining data from numerous core plugs with detailed log-based evaluation, key reservoir properties were quantified. This integrated workflow provides a more reliable representation of the reservoir quality and heterogeneity, enabling a clearer evaluation of the hydrocarbon potential within the Nukhul Formation.

### Petrophysical logs analysis

The primary objectives of well log petrophysical analysis are to determine the net pay thickness and to estimate the volume of hydrocarbons accumulated within the formation. This study centers on the interpretation of the complete set of conventional well logs obtained from the Sidri-14 well, located in the Belayim Land Oil Field within the Gulf of Suez, Egypt. To achieve accurate interpretation and analysis, the Techlog software was employed. This software facilitated the integration of various well logging datasets, enabling a comprehensive evaluation of the petrophysical properties of each unit. The analysis provided insights into lithology discrimination, porosity estimation, and fluid identification and ultimately allowed for the quantification of hydrocarbon-bearing zones across the studied intervals within the Nukhul Formation. Many authors, such as^43–46^, have used the standard cutoff to calculate the different petrophysical parameters.

In this study, the vertical well logging data were analyzed using a suite of conventional tools, including gamma ray, resistivity, sonic, density, and neutron logs. The analysis was carried out for the Sidri-14 well at a depth interval of around 2836 m (TVD). Particular emphasis was placed on the Nukhul Formation, which represents the primary reservoir target in the study area. The calibration of reservoir petrophysical properties was achieved through core-derived measurements^16^.

The shale volume (Vsh) was calculated from the gamma ray log using the standard equation described by^47,48^ and following the younger rocks method^1^. To evaluate the reservoir’s fluid storage capacity, the total porosity was determined, as it reflects the overall pore volume within the rock. A combination of neutron and density logs was employed for this purpose, as recommended by^49^. However, the ability of the reservoir to transmit fluids is governed by effective porosity, which corresponds to the volume of interconnected pores. According to^15,16^, effective porosity was calculated by integrating neutron-derived porosity with density-derived porosity, following the approach of^49^. The hydrocarbon saturation (Shc) was estimated as the complement of water saturation (Sw), i.e., Shc = 1-Sw. The Archie’s relationship^50^was employed to compute Sw, with necessary adjustments applied using the resistivity log data. For this equation, we applied a = 0.98 and m = 2.11 as revealed from the SCAL data analysis. Nabawy et al^51^. state that Archie’s parameters are affected by the shale content, so he recommended importing Archie’s parameters from the SCAL data in case of its application to the shaly sandstone reservoirs, i.e., it is applicable to this type of reservoir^52–54^. Finally, to derive the net-to-gross ratio (NTG) and net pay thickness, standard cutoff criteria were applied: porosity ≥ 10%, shale volume ≤ 35%, and water saturation ≤ 50%. These parameters ensured that only intervals with adequate reservoir quality and hydrocarbon potential were classified as productive zones.

This workflow enabled the subdivision of the reservoir into discrete HFUs, providing a clearer understanding of the heterogeneity, flow performance, and quality distribution within the Nukhul Formation. Such classification enhances reservoir modeling and supports more accurate predictions of productivity^55–57^.

### Core measurements

For the petrophysical investigation, three separate cores were recovered from the Lower Miocene sequence (Sidri-14 well), Nukhul Formation. These cores were subjected to routine core analyses to obtain key reservoir properties, including porosity, permeability (kh and kv), grain and bulk densities, and fluid saturations. The results derived from the core plugs provided direct and reliable measurements of the reservoir characteristics, which were then used to calibrate and validate the well log interpretations.

A total of 215 core plug samples were analyzed. For each sample, porosity was determined under ambient conditions by measuring both the grain volume and the pore volume, allowing for an accurate estimation of the rock’s total pore space. Permeability measurements were also conducted to evaluate the ease with which fluids can flow through the interconnected pore system. Out of the total samples, 22 plug samples were prepared for the SCAL measurements; however, six could not be obtained. These core-derived parameters provided essential inputs for calibrating well log interpretations and characterizing the reservoir quality of the studied intervals. Furthermore, they were used to calibrate and validate the petrophysical interpretations of well logs.

The Nukhul Formation samples were analyzed through a set of reservoir quality parameters that have been extensively utilized in recent petrophysical and reservoir studies^12–14,17,18,21,51^. These parameters include the Reservoir Quality Index (RQI, in μm), the Normalized Porosity Index (NPI, in fractions), the Flow Zone Indicator (FZI, in μm), and the Reservoir Potentiality Index (RPI). Each of these indices provides a quantitative measure for evaluating pore-throat attributes, reservoir heterogeneity, and potential flow performance. The main indices applied in this study include:

#### Reservoir quality index (RQI, μm)

RQI is a measure of the effective pore-throat radius that controls fluid flow within the rock. It is calculated as

1 $$RQI = 0.0314*\sqrt k /\emptyset$$

Where k is permeability (mD) and ∅ is porosity (fraction). Higher RQI values indicate larger effective pore throats and, consequently, better reservoir quality.

#### Normalized porosity index (NPI, fraction)

According to^11^, NPI is defined as the porosity normalization against the grain volume of the system, i.e., the ratio of the void volume to the grain volume of the system.

2 $$NPI = \frac{\emptyset }{1 - \emptyset }$$

#### Flow zone indicator (FZI, μm)

FZI links RQI and NPI to identify Hydraulic Flow Units (HFUs); which is computed as

3 $$FZI = \frac{RQI}{{NPI}}$$

FZI helps in classifying rocks into distinct flow zones with similar pore-throat geometries and flow behaviors.

#### Reservoir potentiality index (RPI)

RPI integrates porosity and permeability into a single parameter to evaluate the overall storage and flow potential of the reservoir rock. It is particularly useful for ranking samples in terms of productivity.

In addition, the Discrete Rock Types (DRT) approach proposed by Shenawi^58^ was applied. This method classifies rocks into groups with similar hydraulic properties using porosity and permeability relationships. It is widely applied by many authors in its modified form^51,53,54^ as shown in the following lines.

4 $$DRT = Round\left( {2*\ln \left( {FZI} \right) + 10.6;1} \right)$$

This classification scheme allows the separation of samples with different porosity values but having the same flow capacity; thereby improving the predictability of reservoir performance and enhancing the reliability of petrophysical models.

By applying the improved stratigraphic modified Lorenz plot (ISML) technique, the reservoir was subdivided into numbers of distinct hydraulic flow units (HFUs). This approach integrates petrophysical measurements with stratigraphic ordering, allowing for the identification of intervals that share similar pore-throat geometries and flow capacities. The method involves plotting cumulative flow capacity (kh) against cumulative storage capacity (∅h) and grouping depths with comparable reservoir rankings into a single HFU^59–61^. This classification improves the understanding of reservoir heterogeneity by distinguishing intervals with high, moderate, and low flow efficiencies, which are critical for production forecasting and reservoir simulation.

The integration between core and log data ensured more accurate quantification of the reservoir quality and heterogeneity within the studied formation.

## Results and discussion

The evaluation of the studied formation was conducted through the integration of well log interpretations with routine core analysis data, providing a comprehensive assessment of reservoir properties. For this study, three cored intervals were obtained from the Nukhul Formation, and a total of 215 core plug samples were analyzed.

### Petrophysical characterization based on well log data

**Fig. 3:**
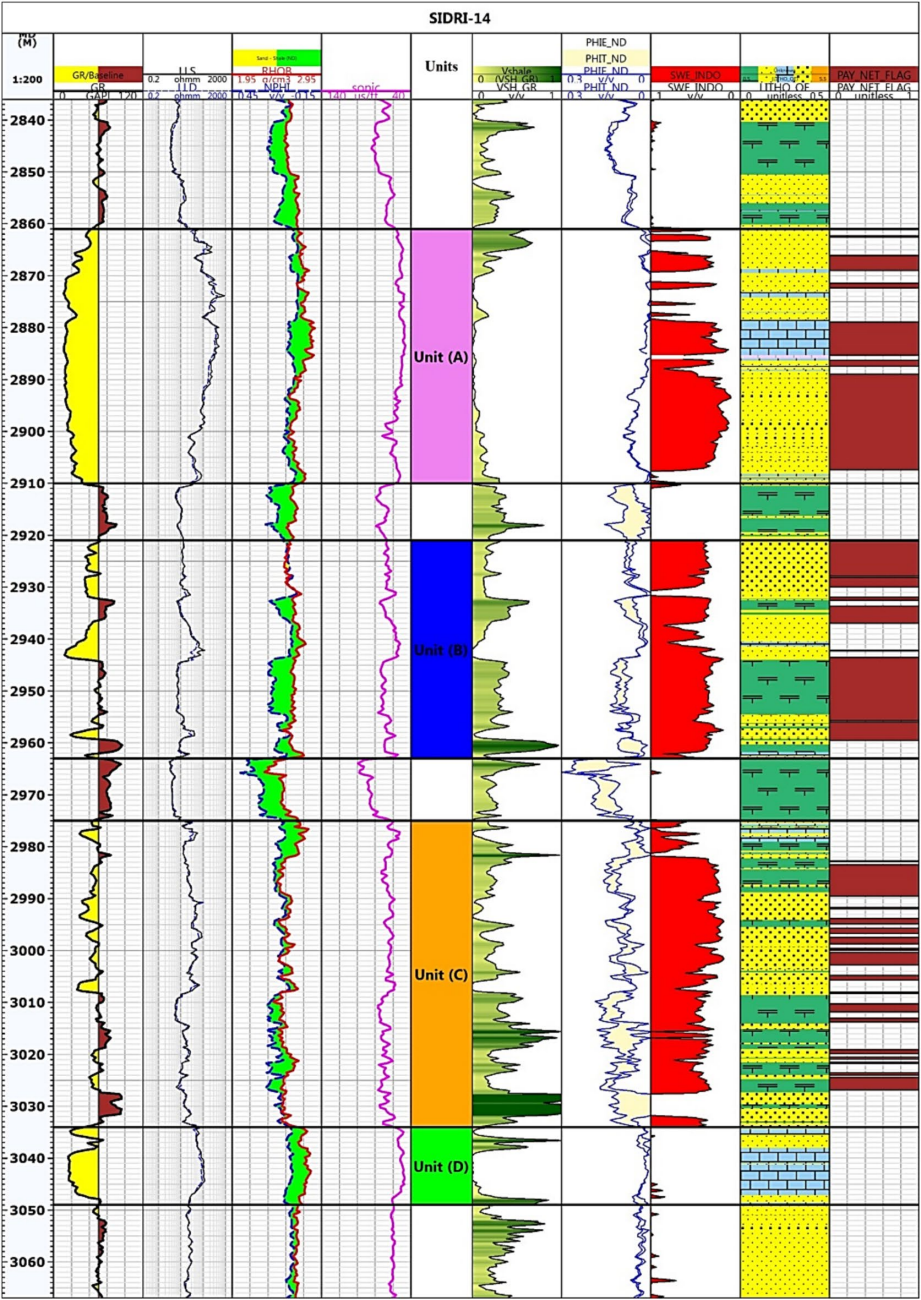
Litho-saturation plot of the studied formation after the discriminating the formation into four units (track 6). Track 7 indicates the shale volume, porosity are presented in track 8, hydrocarbons and water-bearing zones in tracks 9, whereas the net pay zones are represented in track 10.

Figure 3 illustrates the input data and the results of the petrophysical analysis performed using Techlog software. The Nukhul Formation in the Sidri-14 well was subdivided into four units, designated A, B, C, and D, based on lithological and petrophysical variations. Units A, B, and C are composed predominantly of sandstone interbedded with shale and minor limestone layers, reflecting a mixed shallow marine to marginal marine depositional environment. Unit D, in contrast, is largely composed of limestone, with low effective porosity and permeability, rendering it as a non-reservoir.

The average petrophysical properties of each unit, including volume of shale (Vsh), water saturation (Sw), hydrocarbon saturation (Sh), effective porosity (Øe), net pay thickness, and bulk volume of water (BVW), are compiled in Table 1. The results demonstrate that units A, B, and C exhibit good reservoir-quality characteristics, with low porosity averaging 4.3%, 9.5%, and 13.6% for Units A, B, and C, respectively, and good hydrocarbon saturation, and net pay thickness. Coupled with their low permeability (maximum = 13.1 mD), these features classify the productive intervals as unconventional poor reservoirs. In contrast, Unit D does not meet the criteria for a productive reservoir, i.e., relatively low porosity and high water saturation and shale volume. It demonstrates limited reservoir potential due to its high carbonate content and reduced hydrocarbon storage capacity. These distinctions are crucial for reservoir characterization and hydrocarbon evaluation, as they guide the identification of productive zones within the formation.

**Table 1:** The average petrophysical properties of the Nukhul units in Rudeis-Sidri Oil Field.

**Zones**	**Top** **m**	**Bottom** **m**	**Net Pay** **m**	**BVW** **0.0**	**Vsh** **%**	**PhiT** **%**	**SW** **%**	**Hc** **%**
Unit (A)	2861	2910	30.175	0.31	0.074	0.043	0.24	0.76
Unit (B)	2921	2963	28.466	0.952	0.262	0.095	0.354	0.646
Unit (C)	2975	3034	20.422	0.836	0.221	0.136	0.302	0.698
Unit (D)	3034	3049	0.00	0.00	0.00	0.00	0.00	0.00

Where BVW is the bulk volume of water, Vsh is the shale volume, PhiT is the total porosity, Sw and HC are the water and hydrocarbon saturations, respectively.

The core samples were used to determine key petrophysical properties, including porosity, permeability, fluid saturations, and bulk and grain densities, which were subsequently calibrated against the well log data. The combination of core measurements and well logs allowed for a robust characterization of the formation, ensuring accurate identification of productive zones, hydrocarbon distribution, and reservoir quality.

### Reservoir heterogeneity

**Fig. 4:**
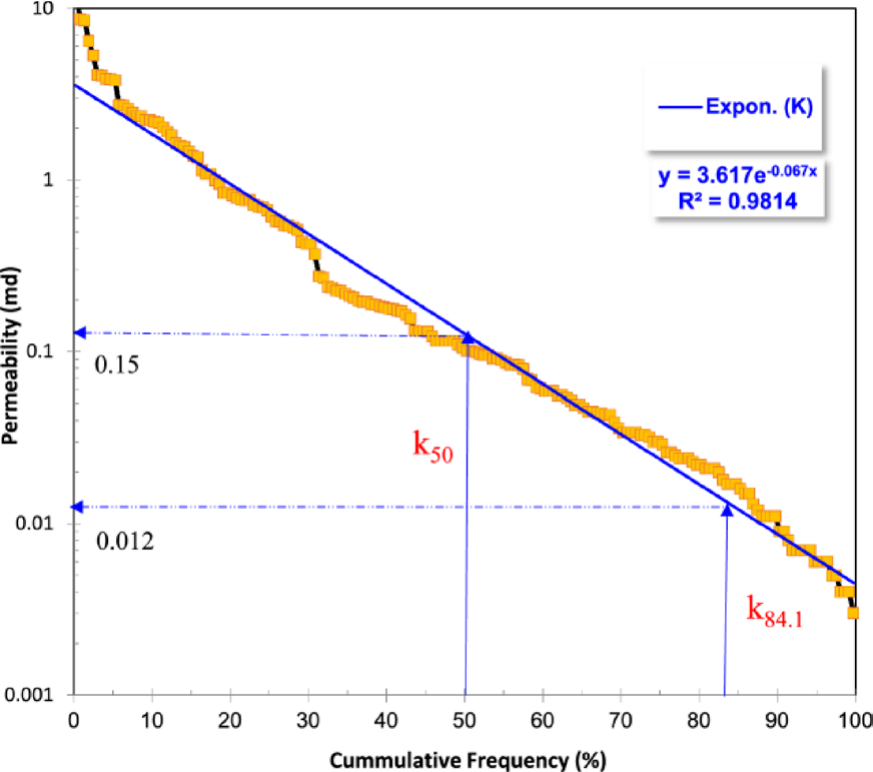
Dykstra-Parson’s technique (Dykstra and Parsons, 1950) for delineating the heterogeneity degree.

The Dykstra-Parsons method (1950)^62^ was applied to evaluate the heterogeneity of the Nukhul reservoir represented by the vertical distribution of the permeability values of the studied sequence, revealing an extremely heterogeneous reservoir with a heterogeneity coefficient (V) of 0.92 (Fig. 4). The permeability values span a wide range, from a minimum of 0.003 mD to a maximum of 13.1 mD, highlighting the significant variability in pore connectivity and flow pathways throughout the Nukhul Formation. This pronounced heterogeneity indicates that fluid movement is highly non-uniform, which may result in preferential flow through higher-permeability zones while bypassing tight intervals, thereby reducing overall hydrocarbon recovery. In addition, the combination of low porosity and low permeability across much of the formation classifies these productive intervals as unconventional reservoirs, requiring specialized production strategies, such as hydraulic fracturing or horizontal drilling, to enhance recovery efficiency. Understanding the permeability distribution and heterogeneity is therefore critical for hydraulic flow unit (HFU) classification, reservoir simulation, and the optimization of production schemes in the Sidri-14 well.

### Reservoir anisotropy

**Fig. 5:**
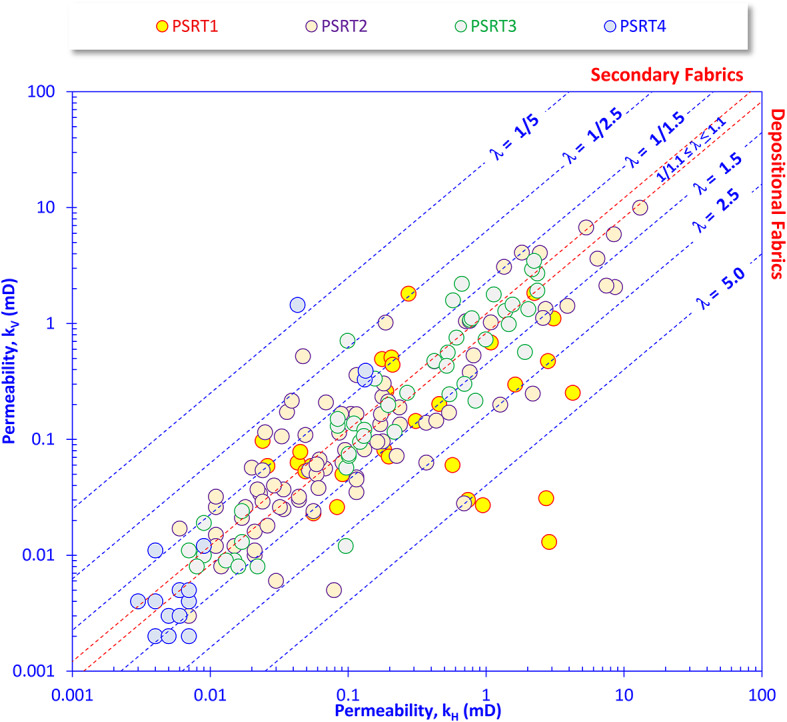
Plotting the permeability in the vertical and horizontal directions versus each other as a technique for revealing the permeability anisotropy.

The reservoir permeability anisotropy refers to the variation in the permeability, horizontally and vertically, at the same point, while the reservoir heterogeneity/homogeneity refers to the permeability variation vertically from bed to bed^12,63^. Therefore, a complementary technique for estimating the permeability variation vertically and horizontally, the permeability anisotropy (λ) of the Nukhul Formation, was estimated as the square root of the ratio between the horizontal and vertical permeability values, indicating that the anisotropy varies greatly from fractions (0.172, extremely secondary anisotropic) to extremely depositional anisotropic (17.25, Table 2.). For the Nukhul Formation, the PSRT1 samples are primarily characterized by slight to extremely depositional fabrics, due to the presence of some shaly streaks, with some moderately secondary fabrics (Fig. 5) due to the presence of some microfractures as explained by many authors^21,54,64^. For the PSRT2-4 samples, they are mostly scattered around the similarity line (λ = 1.0) with some shift toward the secondary fabrics, indicating the dominance of the microfracture patterns. This could be estimated numerically by the variation in anisotropy from 0.300 to 3.085, 0.558 to 3.976, and 0.172 to 2.236 for the PSRT2-4, respectively, with averages equal to 1.078 (isotropic), 1.169 (slightly anisotropic), and 1.013 (isotropic) for the PSRT2-4, respectively (Table 2.).

**Table 2:** Petrophysical properties and reservoir quality parameters of the Nukhul’s PSRTs in Rudeis-Sidri Oil Field, Gulf of Suez.

PSRTs		Bulk Density	Grain Density	Porosity %	k_H_ mD	k_V_ mD	λ_k_ 0.00	RQI μm	FZI μm	DRT 0.00	R_35_ μm	So %	Sw %
PSRT1 (N = 30)	Min	2.505	2.740	1.60	0.024	0.013	0.389	0.038	2.081	12.1	0.401	1.20	19.00
	Max	2.911	2.980	8.90	4.249	1.834	17.25	0.229	4.084	13.4	2.344	21.40	96.30
	Average	2.717	2.823	3.79	0.883	0.321	2.793	0.110	2.749	12.6	1.080	8.16	42.77
PSRT2 (N = 90)	Min	2.263	2.620	1.30	0.006	0.003	0.300	0.020	0.762	10.1	0.179	0.00	17.60
	Max	2.888	2.980	16.80	13.099	9.974	3.085	0.277	2.062	12.0	2.139	41.20	97.60
	Average	2.642	2.787	5.24	0.882	0.639	1.078	0.069	1.350	11.1	0.573	9.41	47.64
PSRt3 (N = 53)	Min	2.295	2.620	2.60	0.007	0.008	0.558	0.014	0.301	8.2	0.089	0.00	36.80
	Max	2.834	2.910	15.30	2.354	3.469	3.975	0.126	0.756	10.0	0.880	38.30	97.80
	Average	2.490	2.735	8.98	0.554	0.595	1.169	0.056	0.545	9.3	0.386	13.92	59.09
PSRT4 (N = 17)	Min	2.410	2.710	4.20	0.003	0.002	0.172	0.008	0.157	6.9	0.049	0.00	43.90
	Max	2.625	2.760	11.40	0.134	1.446	2.236	0.034	0.278	8.0	0.207	21.90	98.00
	Average	2.573	2.737	5.98	0.025	0.148	1.013	0.014	0.215	7.5	0.087	3.25	86.27

Where k_H_ and k_V_ are the horizontal and vertical permeability, respectively; λ_k_is the permeability anisotropy; RQI and FZI are the reservoir quality index and the flow zone indicator of Amaefule et al^11^. DRT is the discrete rock type of Shenawi et al^55^. R_35_is the effective pore sizes of Winland et al^56^.

This high variation in the reservoir anisotropy from extremely secondary to extremely depositional fabrics, in addition to the high heterogeneity values, could be attributed to the variation in the depositional environmental depths with different shale flux and the structural control of the various events that affected the depositional sequences during the Miocene^65,66^.

### Rock typing based on core data

**Fig. 6:**
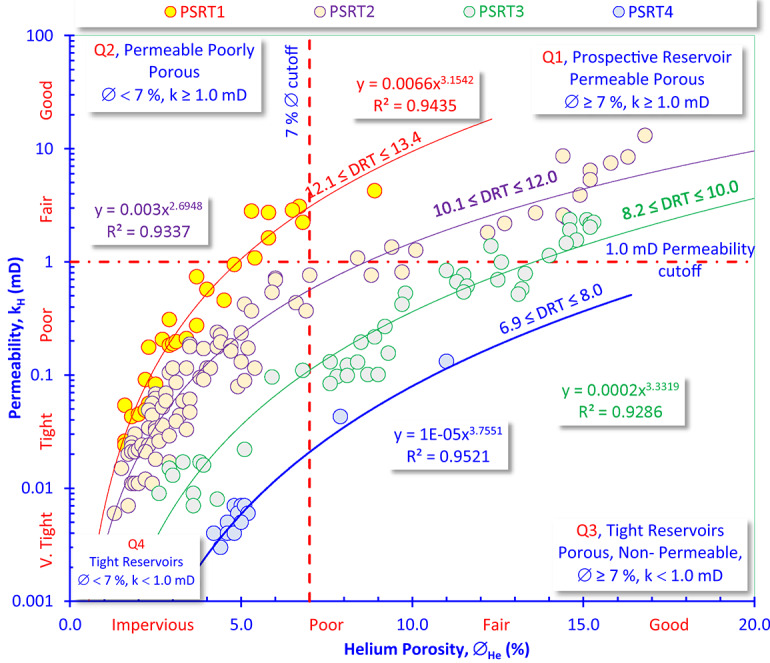
Plotting the horizontal permeability of the Nukhul Formation in Rudeis-Sidri Field versus the helium porosity supported by the DRT values as a tool of rock typing.

Figure 6 is a cross-plot of helium porosity (∅_He_, in %) versus permeability (k_H_, in mD); it highlights the strong heterogeneity of the reservoir system. The plot is divided into four main reservoir quality quadrants (Q1–Q4) based on porosity and permeability cutoffs. Two cutoff values are applied: Porosity cutoff at 7%, differentiating “good to fair” porous intervals from “poor to very tight” porous intervals. Permeability cutoff at 1.0 mD, distinguishing permeable units from non-permeable tight rocks. The data points are color-coded into petrophysical rock types (PSRT1–PSRT4), each represented by a regression line that captures the porosity–permeability trend. The fitted power-law functions yield strong correlation coefficients (R^2^ ranging from 0.9286 to 0.9521), reflecting well-defined relationships between porosity and permeability within each rock type. PSRT1 and PSRT2 (better quality) correspond to Q1–Q2, while PSRT3 and PSRT4 correspond to Q3–Q4 (tight rocks). Table 2. shows the petrophysical properties and reservoir quality parameters of the Nukhul’s PSRTs in the studied formation.

Q1 quadrant (prospective reservoirs) represents the best reservoir quality, where rocks show both high porosity and permeability. These intervals are prospective and can contribute significantly to hydrocarbon production. Q2 (permeable but poorly porous rocks) and Q3 quadrants (porous but non-permeable) indicate reservoir heterogeneity: either permeability exists without sufficient storage capacity (Q2), or porosity exists without adequate flow capacity (Q3). Both require special completion techniques to exploit effectively. The Q4 quadrant (tight reservoirs) represents non-reservoir or extremely tight intervals with negligible contribution to flow. The majority of the Sidri-14 samples fall into Q3 and Q4 quadrants, indicating that the Nukhul Formation is generally a low-permeability, tight reservoir.

### Implications of the effective pore radius (R_35_)

**Fig. 7:**
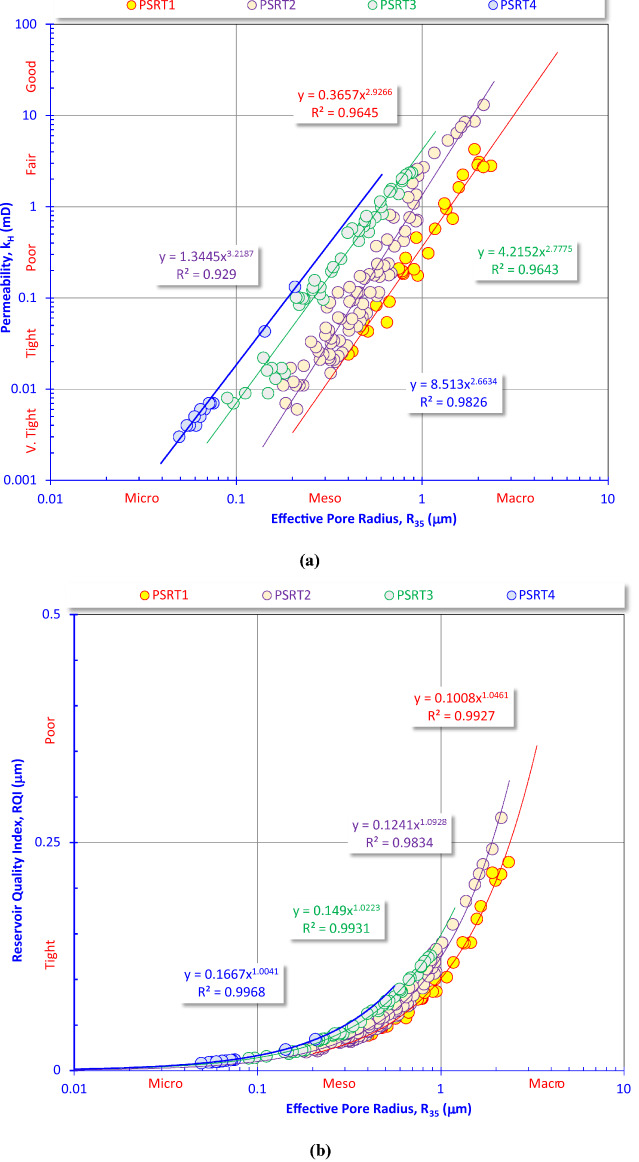
Plotting the effective pore throat radius versus (**a**) permeability and (**b**) reservoir quality index.

Figure 7a is a log–log cross-plot between permeability (k, in mD) versus effective pore radius (R₃₅, in microns) widely used to evaluate reservoir quality and flow potential. This plot proves that permeability in the studied formation is almost entirely controlled by pore throat size (R₃₅). Many samples fall in tight to poor reservoir zones (<1 mD); this means the rocks behave like unconventional reservoirs, needing stimulation such as hydraulic fracturing to produce. Only some points (upper right, larger R₃₅) reach fair to good reservoir quality (>10 mD). The high R^2^ values (0.93–0.98) mean permeability is strongly controlled by effective pore radius. This figure shows that permeability in these rocks is strongly controlled by effective pore throat radius. Among the four rock types, PSRT1 has the best reservoir quality, while PSRT4 is the tightest. Overall, the reservoir is dominated by tight to poor quality rocks, typical of unconventional reservoirs with limited intervals showing fair to good flow potential. While cross-plot (Fig. 7b) illustrates the strong empirical relationship between effective pore-throat radius (R_35_) and the Reservoir Quality Index (RQI in μm) across the four reservoir types (PSRT1–PSRT4). The RQI values increase systematically with larger R_35_ values, indicating that pore-throat size exerts the dominant control on reservoir quality in the studied Nukhul Formation intervals. Most data points fall within the tight to poor quality domain (0.25 R_35_ < 1 μm). This confirms that the studied reservoir intervals are dominated by meso- and macropore classifications, which severely restrict permeability and fluid mobility. The small throat radii suggest strong diagenetic modification (e.g., cementation, compaction, clay plugging). PSRT1 and PSRT2 retain relatively better reservoir potential compared to PSRT3 and PSRT4. This data confirms that the studied intervals should be classified as unconventional, tight reservoirs that require stimulation to achieve economic production.

### Reservoir quality parameters

**Fig. 8:**
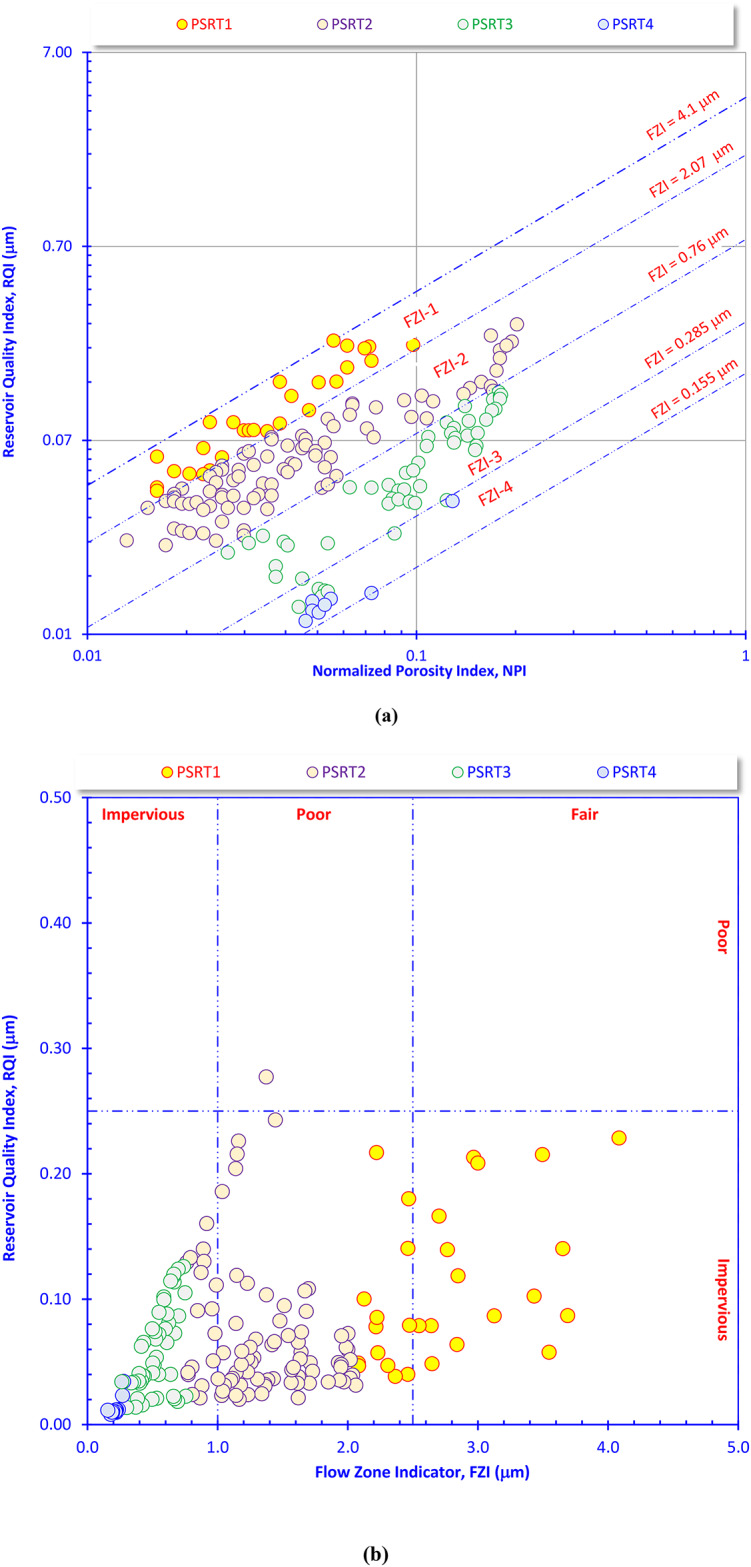
Plotting the RQI versus (**a**) the NPI, and (**b**) the FZI as a technique for quality check and reservoir quality ranking.

Figure 8a is a tool for rock typing and reservoir characterization. The figure demonstrates that reservoir heterogeneity is controlled by pore-throat architecture rather than porosity alone**.** It links porosity and permeability into flow units using FZI (values of FZI ≈ 0.15, 0.285, 0.76, 2.07, and 4.1 µm, respectively, from 4 to 1). The dataset divides into several HFUs. Points falling along the same FZI line belong to the same Hydraulic Flow Unit, meaning they share similar pore geometry and flow behavior even if porosity varies, showing clear separation in reservoir quality among the four sample groups (PSRT1–PSRT4). Each color represents a distinct HFU (rock type). PSRT1 shows the highest RQI and highest FZI1**,** wide pore-throat sizes, and the best permeability and flow capacity. This rock type corresponds to HFU-1 (best reservoir quality). PSRT2 samples have moderate RQI and FZI values, i.e., constitute good flow units. This rock type is equivalent to HFU-2. The PSRT3 has lower RQI and smaller pore throats and limited flow capacity. This rock type corresponds to HFU-3. Finally, the PSRT4 has the lowest RQI and FZI, indicating that it is a very tight rock type. This rock type has poor to negligible flow (often non-net). Finally, the HFU-1 and HFU-2 represent the most effective flow units, and therefore they should be prioritized for perforation placement, are the preferred targets for hydraulic fracture stages, while lower HFUs (3 and 4) should be avoided to prevent ineffective stimulation and reduced economic return. Perforation placement, and hydraulic fracture stages should be preferentially targeted within HFU-1 and, to a lesser extent, HFU-2, to maximize access to high-quality flow conduits and improve overall production performance. The RQI–NPI cross-plot (Fig. 8b) confirms a strong heterogeneity in the Nukhul Formation, where PSRT1 has the highest FZI values and moderate to relatively high RQI; it represents HFU-1 which is the best flow unit with well-connected pore systems. PSRT2 has intermediate FZI and RQI; it corresponds to HFU-2, a secondary but still productive flow unit. PSRT3 has low FZI and low RQI, it represents HFU-3, characterized by poor pore connectivity and limited flow. PSRT4 has very low FZI and RQI; it is tight and impervious rock type with negligible reservoir potential (non-net).

### Buckle’s plot

**Fig. 9:**
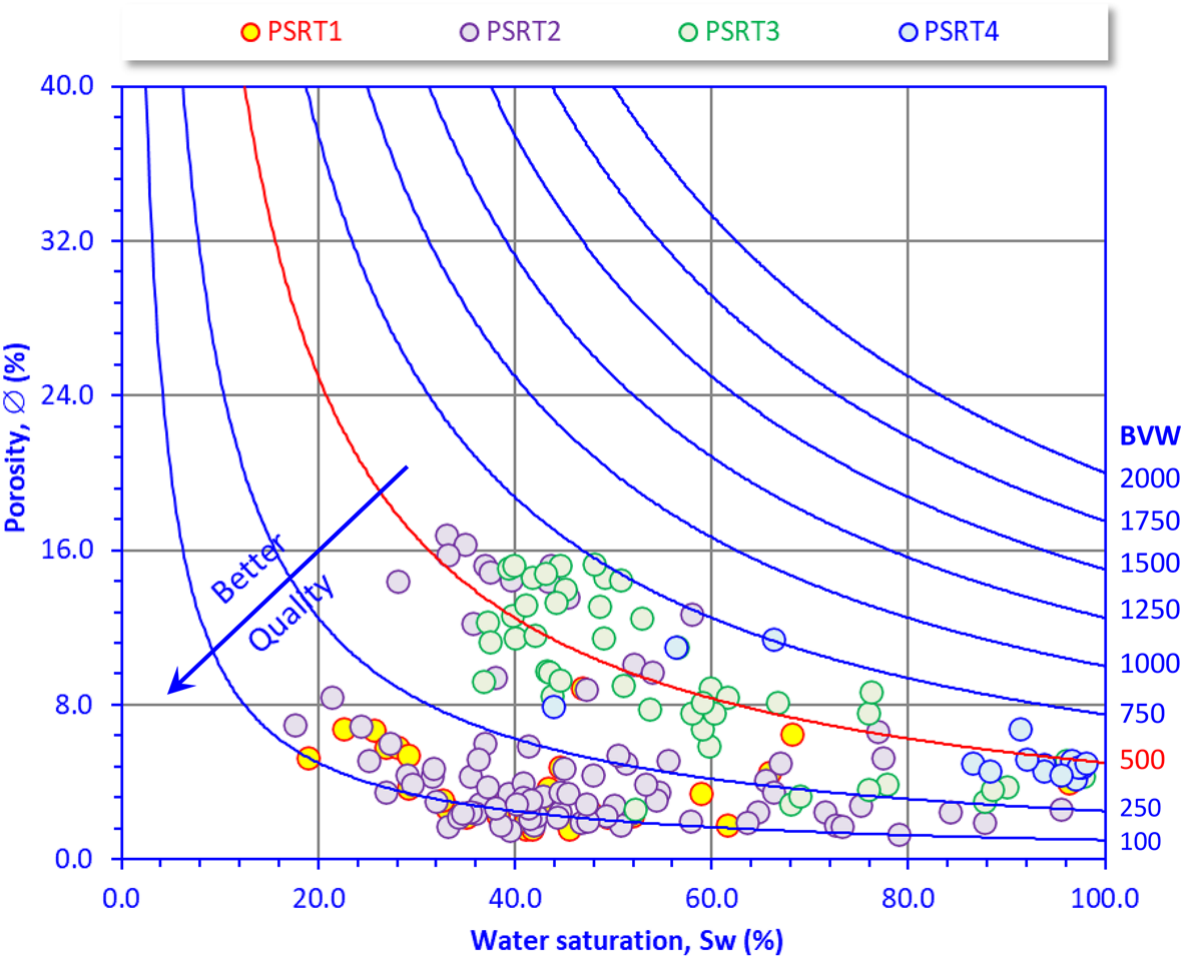
Buckles Plot (Buckles, 1965) for delineating the bulk volume of water (BVW).

The porosity–water saturation crossplot (Fig. 9) is a diagnostic plot used to identify and estimate the retained bulk volume of water (BVW), i.e., reservoir quality. Low BVW values (< 500) usually indicate oil production with zero water cut, while higher BVW values indicate more water-filled pores and poorer reservoir quality. Also, the higher the BVW, the lower the reservoir quality, and vice versa^66,67^. The studied samples mostly fall in the low to moderate porosity range with moderate-to-high water saturation, indicating tight to sub-commercial reservoir quality with limited hydrocarbon potential. PSRT1 and PSRT2 are clustered at low porosity (2–8%) and moderate to high water saturation (20–60%), showing tight, poorer quality reservoirs. PSRT3 samples are characterized by moderate porosity (8–16%) with variable water saturation (30–70%), suggesting fair reservoir zones with mixed quality. This classification is better than PSRT1-2, it falls into the better quality zone. PSRT4 samples are scattered at low to moderate porosity with high Sw (>60%), indicating non-reservoir or water-bearing intervals.

However, most of the PSRTs samples have BVW less than 500, while the other samples are clustered slightly higher than the 500 value; therefore, most of the Nukhul reservoir samples refer to oil production with no water, especially for the PSTR1-2 samples, while the PSRT3-4 produce oil with low water content.

### Flow units discrimination

**Fig. 10:**
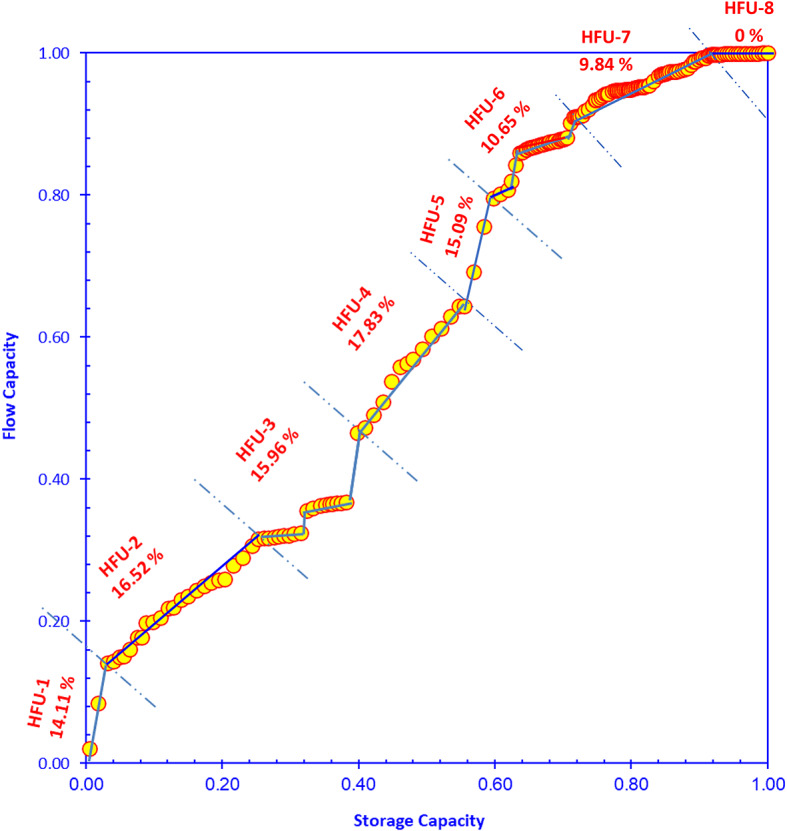
Discriminating the cored interval into a set of hydraulic flow units (HFUs) using the improved stratigraphic Lorenz Plot technique (ISML)^60^.

The reservoir is divided into eight Hydraulic Flow Units (HFUs), as shown in Figure 10. The slope of each segment reflects the relative contribution of each HFU to fluid flow, where steeper segments indicate higher flow efficiency, higher permeability, larger pore-throat sizes and gentler slopes indicate poorer flow performance.

HFU-1 and HFU-2 appear at low storage capacity but show relatively steep slopes, contributing 14.11% and 16.52% of the total flow capacity, respectively. This indicates that despite their limited storage volume, these units are highly efficient flow conduits. These HFUs are characterized by the highest RQI and FZI values, wide pore-throat sizes, high permeability, and excellent flow capacity. Consequently, they represent the best reservoir quality and most effective flow units as referred by some authors^68–70^.

HFU-3 to HFU-5 exhibit gentler slopes and contribute 15.96%, 17.83% and15.09% of the flow capacity, respectively, which are characterized by lower RQI, smaller pore throats, and reduced permeability. Their subdued contribution to flow suggests intermediate flow performance, and they are commonly classified as moderate-quality reservoir intervals. In contrast, HFU-6 and HFU-7 have poor to marginal flow performance with poor reservoir quality and low flow efficiency. HFU-8 contributes negligibly to flow, consistent with its zero flow percentage. This means they are poor quality and essentially non-reservoir rock. This distribution shows heterogeneous reservoir quality, with fluid flow dominated by a few high-quality flow units.

By applying these methodologies, the studied Sidri-14 well can be divided into discrete hydraulic flow units. This classification provides a continuous distribution of HFUs across both cored and uncored sections of the reservoir, enabling improved characterization of reservoir heterogeneity and flow performance within the Nukhul Formation as recommended by many authors^71–73^.

The study focuses on the Nukhul Formation in the Sidri-14 well, Gulf of Suez, integrating core and log data to assess reservoir quality and heterogeneity. The Nukhul consists mainly of sandstone, shale, and limestone deposited in shallow-marine to deltaic settings. Porosity–permeability relationships, BVW plots, and flow unit analysis reveal significant vertical and lateral variations in reservoir quality, controlled by depositional facies and diagenesis, which influence hydrocarbon storage and flow capacities^74^.

## Conclusions

The integrated analysis of well logs and core data from the Sidri-14 well in the Rudeis–Sidri Oil Field has provided a comprehensive characterization of the Nukhul Formation. Petrophysical evaluation, supported by core samples and well log analysis, subdivided the formation into four reservoir units (A, B, C, and D). Units A, B, and C represent mixed sandstone–shale successions, while Unit D is mainly limestone with no reservoir potential. The average effective porosity of Units A, B, and C is 4.3%, 9.5%, and 13.6%, respectively, while permeability values range widely from 0.003 to 13.1 mD, with a heterogeneity coefficient (V) of 0.92 that classifies the formation as highly heterogeneous. Hydrocarbon saturation shows moderate variability, while water saturation (Sw) frequently exceeds 50%, limiting net pay, whereas Unit D, being carbonate-dominated, shows non-net reservoir potential. The porosity–permeability relationship (0.93 ≤ R^2^ ≤ 0.98) confirms permeability is strongly controlled by effective pore-throat size (R_35_), with most samples falling in the tight/poor quality domains (0.25 R_35_ < 1.0 μm), requiring stimulation for economic recovery.

Bulk volume water plots indicate that the majority of intervals fall within low-to-moderate porosity and high water saturation zones, suggesting sub-commercial reservoir quality. These classify the productive zones as unconventional tight reservoirs.

PSRT1 and PSRT2 show the highest RQI and highest FZI1**,** wide pore-throat sizes, and the best permeability and flow capacity. These rock types correspond to HFU-1 and HFU-2 (best reservoir quality). PSRT3 and PSRT4 have lower RQI and smaller pore throats, and limited flow capacity. These rock types correspond to HFU-3 and HFU-4, and they have poor to negligible flow (often non-net). Finally, HFU-1 and HFU-2 represent the most effective flow units, and therefore they should be prioritized for perforation placement, i.e., they are preferred targets for hydraulic fracture stages, while the other HFUs (3 and 4) should be avoided to prevent ineffective stimulation and reduced economic return.

Hydraulic Flow Unit (HFU) classification subdivides the reservoir into 8 HFUs**,** where HFU-1 and HFU-2 contribute the largest portion of flow (14.11% and 16.52% of total flow capacity) and equivalent to PSRT1 and PSRT2**,** which are characterized by the highest RQI and FZI values, wide pore-throat sizes, high permeability, and excellent flow capacity. Consequently, they represent the best reservoir quality and most effective flow units. HFU-3 to HFU-5 exhibit gentler slopes and contribute 15.96%, 17.83% and 15.09% of the flow capacity, respectively. These units correspond to PSRT3 and PSRT4, which are characterized by lower RQI, smaller pore throats, and reduced permeability. Their subdued contribution to flow suggests intermediate flow performance, and they are commonly classified as moderate-quality reservoir intervals. The majority of the Sidri-14 samples fall into PSRT3 and PSRT4 which are equivalent to Q3 and Q4 quadrants, indicating that the Nukhul Formation is generally a low-permeability, tight reservoir. HFU-6 and HFU-7 have poor to marginal flow performance with poor reservoir quality and low flow efficiency. In contrast, HFU-8 contributes negligible flow (0%), highlighting non-reservoir rock.

Overall, the Nukhul Formation in the studied well should be regarded as an unconventional, heterogeneous tight reservoir, where production will rely on targeting the limited high-quality flow units and applying advanced recovery techniques such as hydraulic fracturing and horizontal drilling to achieve economic production. Completion activities should focus primarily on HFU-1 and HFU-2 to achieve optimal reservoir connectivity and production efficiency.

## Data Availability

Data will be available on reasonable request by contacting the corresponding author: [Marwa_epri@yahoo.com](mailto:Marwa_epri@yahoo.com).

## Declarations

### Permission of data

Permission and consent were obtained from the Egyptian General Petroleum Corporation for conducting the field study in the Nukhul Formation, Rudeis-Sidri Oil Field, Egypt.

## References

[1] Radwan, A. E., Trippetta, F., Kassem, A. A. & Kania, M. Multi-scale characterization of unconventional tight carbonate reservoir: Insights from October oil filed, Gulf of Suez Rift Basin, Egypt. *J. Pet. Sci. Eng.* **197**, 107968. 10.1016/j.petrol.2020.107968 (2021).

[2] Metwally, A., Refaat, A., El-Gawad, E. A., Fathy, M. & Mosad, M. Fracture recognition and characterization of the unconventional igneous intrusion reservoir in Rudeis–Sidri Field, Gulf of Suez, Egypt. *J. Geol. Geophys.* **11**, 017. 10.35248/2381-8719-22.11.1012 (2022).

[3] EGPC (Egyptian General Petroleum Corporation), (1996): Gulf of Suez Oil Fields (A Comprehensive Overview). Egyptian General Petroleum Corporation, Cairo, p. 736pp.

[4] Zahra, H. S. & Nakhla, A. M. Structural interpretation of seismic data of Abu Rudeis-Sidri area, Northern Central Gulf of Suez, Egypt. *NRIAG J. Astron. Geophys.* **5**(2), 435–450. 10.1016/j.nrjag.2016.09.002 (2016).

[5] Elmaadawy, K. G., Bayan, F. M., El-Shayeb, H.M.; (2021): Source rock maturity and hydrocarbon potential of Abu Rudeis‑Sidri area, central province, Gulf of Suez, Egypt. Arabian Journal of Geosciences. 10.1007/s12517-021-09140-6.

[6] Fathy, D., Lee, E. Y., Xiang, X., Fathi, E. & Sami, M. Petrophysical properties of the middle Miocene sediments on the central Gulf of Suez, Egypt. *Front. Earth Sci.* **13**, 1592041. 10.3389/feart.2025.1592041 (2025).

[7] De Ros, L.F., Goldberg, K.; (2007): Reservoir petrofacies: a tool for quality characterization and prediction, part 1. In: AAPG, Annual Convention and Exhibition, Long Beach.

[8] Tiab, D., Donaldson, E.C.; (2016): Petrophysics: Theory and Practice of Measuring Reservoir Rock and Fluid Transport Properties, 4th edition. Elsevier / Gulf Professional Publishing, ISBN: 978-0128031889. 10.1016/C2014-0-03707-0

[9] Kassab, M. A., Abdou, A. A., El Gendy, N. H., Shehata, M. G. & Abuhagaza, A. A. Reservoir characteristics of some Cretaceous sandstones, North Western Desert, Egypt. *Egypt. J. Pet.* **26**, 391–403. 10.1016/j.ejpe.2016.05.011 (2017).

[10] El Sawy, M. Z., Abuhagaza, A. A., Nabawy, B. S. & Lashin, A. Rock typing and hydraulic flow units as a successful tool for reservoir characterization of Bentiu–Abu Gabra sequence, Muglad basin, Southwest Sudan. *J. Afr. Earth Sci.* **171**, 103961. 10.1016/j.jafrearsci.2020.103961 (2020).

[11] Amaefule, J., Altunbay, M., Tiab, D., Kersey, D., Keelan, D.; (1993): Enhanced reservoir description, using core and log data to identify hydraulic (flow) units and predict permeability in uncored intervals/wells. Houston, Texas. In: SPE Annual Technical Conference and Exhibition. SPE-26436-MS. 10.2118/26436-MS

[12] Nabawy, B. S., Khalil, H. M., Fathy, M. S. & Ali, F. Impacts of microfacies type on reservoir quality and pore fabric anisotropy of the Nubia sandstone in the central Eastern Desert, Egypt. *Geol. J.* **55**(6), 450–4524. 10.1002/gj.3690 (2020).

[13] Nabawy, B. S., Lashin, A. & Barakat, M. . Kh. . Implementation of lithofacies and microfacies types on reservoir quality and heterogeneity of the Late Cretaceous Upper Bahariya Member in the Shurouk Field, Shoushan Basin, North Western Desert, Egypt. *J. Asian Earth Sci.* **224**, 105014. 10.1016/j.jseaes.2021.105014 (2022).

[14] Elmahdy, M., Radwan, A. A., Nabawy, B. S., Abdelmaksoud, A. & Nastavkin, A. V. Integrated geophysical, petrophysical and petrographical characterization of the carbonate and clastic reservoirs of the Waihapa Field, Taranaki Basin, New Zealand. *Mar. Pet. Geol.* **151**, 106173. 10.1016/j.marpetgeo.2023.106173 (2023).

[15] Radwan, A. E., Abudeif, A. M. & Attia, M. M. Investigative petrophysical fingerprint technique using conventional and synthetic logs in siliciclastic reservoirs: A case study, Gulf of Suez basin, Egypt. *J. Afr. Earth Sci.* **167**, 103868. 10.1016/j.jafrearsci.2020.103868 (2020).

[16] Radwan, A. E., Nabawy, B. S., Kassem, A. A. & Hussein, W. S. Implementation of rock typing on waterflooding process during secondary recovery in oil reservoirs: A case study, El Morgan Oil Field, Gulf of Suez, Egypt. *Nat. Resour. Res.* **30**(Suppl. 2), 1667–1696. 10.1007/s11053-020-09806-0 (2021).

[17] El Sharawy, M. S. & Nabawy, B. S. Determining the porosity exponent m and lithology factor a for sandstones and their control by overburden pressure: A case study from the Gulf of Suez, Egypt. *AAPG Bull.* **102**(9), 1893–1910. 10.1306/03141817262 (2018).

[18] Mebrouki, N., Nabawy, B., Hacini, M. & Abdel-Fattah, M. I. Deciphering the implication of microfacies types and diagenesis on the reservoir quality of the Cambrian sequence in Hassi Messaoud Field, Algeria. *Mar. Pet. Geol.*10.1016/j.marpetgeo.2023.106650 (2024).

[19] Maglio-Johnson, T.; (2000): Flow Unit Definition Using Petrophysics in a Deep Water Turbidite Deposit, Lewis Shale, Carbon County. Colorado School of Mines, Wyoming. Publishing M.Sc. thesis.

[20] Nabawy, B. S., Rashed, M. A., Mansour, A. S. & Afify, W. S. M. Petrophysical and microfacies analysis as a tool for reservoir rock typing and modeling: Rudeis Formation, off-shore October Oil Field, Sinai. *Mar. Pet. Geol.* **97**, 260–276. 10.1016/j.marpetgeo.2018.07.011 (2018).

[21] Nabawy, B. S., Mansour, A. S., Rashed, M. A. & Afify, W. S. M. Implementation of sedimentary facies and diagenesis on the reservoir quality of the Aquitanian-Burdigalian Rudeis Formation in the Gulf of Suez, Egypt: A comparative surface and subsurface study. *Geol. J.* **55**(6), 4543–4563. 10.1002/gj.3683 (2020).

[22] Fallah-Bagtash, R. H., Adabi, M. H., Nabawy, B. S., Omidpour, A. & Sadeghi, A. Integrated petrophysical and microfacies analyses for a reservoir quality assessment of the Asmari Dolostone sequence in the Khesht Field, SW Iran. *J. Asian Earth Sci.*10.1016/j.jseaes.2021.104989 (2022).

[23] Ayyad, H. M. et al. Multifactorial controls on carbonate–clastic sedimentation in rift basins: Integrated foraminiferal, sequence stratigraphic, and petrophysical analysis, Gulf of Suez, Egypt. *Minerals* **15**(8), 864. 10.3390/min15080864 (2025).

[24] Reda, M. et al. Hydrocarbon reservoir characterization in the challenging structural setting of Southern Gulf of Suez: Synergistic approach of well log analyses and 2D seismic data interpretation. *Preprints*10.20944/preprints202401.0529.v1 (2024).

[25] Alsharhan, A. S. Petroleum geology and potential hydrocarbon plays in the Gulf of Suez rift basin, Egypt. *AAPG Bull.* **87**(1), 143–180 (2003).

[26] El Atfy, H., Brocke, R. & Uhl, D. Age and paleoenvironment of the Nukhul Formation, Gulf of Suez, Egypt: Insights from palynology, palynofacies and organic geochemistry. *GeoArabia* **18**(4), 137–174. 10.2113/geoarabia1804137 (2013).

[27] Richardson, M. & Arthur, M. A. The Gulf of Suez-Northern Red Sea Neogene Rift: A quantitive basin analysis. *Mar. Pet. Geol.* **5**, 247e–2270 (1988).

[28] Peijs J.AM.M., Bevan T.G, Piombino J.T.; (2012): The Gulf of Suez rift basin”, Regional Geology and Tectonics: Phanerozoic Rift Systems and Sedimentary Basins. Elsevier 164–194. https:// doi. org:10. 1016/ b978-0- 444- 56356-9. 00007-9.

[29] El Beialy, S.Y., Mahmoud, M.S., Ali, A.S.; (2005): Insights on the age, climate and depositional environments of the rudeis and kareem formations, GS-78-1 well, gulf of suez, Egypt: a palynological approach. Review Rev. Espa~nola Micropaleontol. 37 (2), 273e289.

[30] Soliman, A. C., Oric, S., Head, M. J., Piller, W. E. & El Beialy, Y. S. Lower and Middle Miocene biostratigraphy, Gulfof Suez, Egypt based on dinoflagellate cysts and calcareousnannofossils. *Palynology* **36**(1), 38–79. 10.1080/01916122.2011.633632 (2012).

[31] Hughes, G. W., Abdine, S. & Girgis, M. H. Miocene biofacies development and geological history of the Gulf of Suez, Egypt. *Mar. Pet. Geol.* **9**, 2–28. 10.1016/0264-8172(92)90002-V (1992).

[32] Schutz, K.L.; (1994): In: Landon, S.M. (Ed.), Structure and Stratigraphy of the Gulf of Suez, Egypt: Interior Rift Basins, vol. 59. AAPG Memoir, pp. 57–96. 10.1306/M59582C3

[33] Pocknall, D.T., Krebs, W.N., Tawfik, E., Ahmed, A.A.; (1999): Pliocene climate and depositional environments, Gulf of Suez, Egypt: evidence from palynology and diatoms. Rev. Pliocene Time change. Am. Assoc. Stratigr. Palynol. Found. 163-171.

[34] Abu Al-Atta, M., Ibrahim Issa, G., Ahmed, M. A. & Mustafa Afife, M. Source rock evaluation and organic geochemistry of Belayim marine oil field, Gulf of Suez, Egypt. *Egypt. J. Pet.* **23**(3), 285–302. 10.1016/j.ejpe.2014.08.005 (2014).

[35] Afife, M. M., Abu Al-Atta, M., Ahmed, A. M. & Issa, I. G. Thermal maturity and hydrocarbon generation of the Dawi Formation, Belayim Marine Oil Field, Gulf of Suez, Egypt: A 1D basin modeling case study. *Arab. J. Geosci.* **9**(5), 1–31. 10.1007/s12517-016-2320-2 (2016).

[36] Kassem, A. A. et al. Petrographic and diagenetic study of siliciclastic Jurassic sediments from the northeastern margin of Africa: Implication for reservoir quality. *J. Pet. Sci. Eng.*10.1016/j.petrol.2020.108340 (2021).

[37] Radwan, A. E. Modeling the depositional environment of the sandstone reservoir in the Middle Miocene Sidri Member, Badri Field,Gulf of Suez Basin, Egypt: Integration of Gamma-Ray log patterns and petrographic characteristics of lithology. *Nat. Resour. Res.*10.1007/s11053-020-09757-6 (2021).

[38] Farouk, S., Sen, S., Pigott, J. D. & Sarhan, M. A. Reservoir characterization of the Middle Miocene Kareem Sandstones, Southern Gulf of Suez Basin, Egypt. *Geomech. Geophys. Geo-energ. Geo-resour.* **8**, 130. 10.1007/s40948-022-00437-8 (2022).

[39] Barakat, M., Reda, M., Gamvroula, D. E., Ondrak, R. & Alexakis, D. E. Investigating attributes of oil source rocks by combining geochemical approaches and basin modelling (Central Gulf of Suez, Egypt). *Resources* **14**, 114. 10.3390/resources14070114 (2025).

[40] El-Azabi, M. H. Sedimentary evolution of the Miocene syn-rift marginal and deep marine succession, Gulf of Suez, Egypt: A review. *Earth Sci. Rev.* **258**, 104944 (2024).

[41] Elkady, H.H., Ahmed, A.S.S., Mohamed, M.F., Taher, M.T.; (2015): Core-Log Integrated Formation Evaluation and Application of Flow Unit Concept at Rudeis-Sidri Field, Gulf of Suez, Egypt.” International Journal of Innovative Science, Engineering & Technology, V.2.

[42] Farouk, Sh. . et al. Assessment of the petrophysical properties and hydrocarbon potential of the Lower Miocene Nukhul Formation in the Abu Rudeis-Sidri Field. *Geomech. Geophys. Geo-energ. Geo-resour.* **9**, 36. 10.1007/s40948-023-00572-w (2023).

[43] Deng, W. A., Kim, T. & Jang, S. Seismic attributes for characterization of a heavy-oil shaly-sand reservoir in the Muglad Basin of South Sudan. *Geosci. J.* **22**, 1027–1039. 10.1007/s12303-018-0006-3 (2018).

[44] Jumat, N., Shalaby, M. R., Haque, A. E., Islam, M. A. & Lee Hoon, L. Geochemical characteristics, depositional environment, and hydrocarbon generation modeling of the Upper Cretaceous Pakawau Group in Taranaki Basin, New Zealand. *J. Pet. Sci. Eng.* **163**, 320e–3339. 10.1016/j.petrol.2017.12.088 (2018).

[45] Qadri, S. M. T., Islam, M. A., Shalaby, M. R. & El-Aal, A. K. A. Reservoir quality evaluation of the Farewell Sandstone by integrating sedimentological and well log analysis in the Kupe South Field, Taranaki Basin-New Zealand. *J. Pet. Explor. Prod. Technol.* **11**, 11–31. 10.1007/s13202-020-01035-8 (2020).

[46] Shalaby, M. R., Binti Sapri, S. H. & Islam, M. A. Integrated reservoir characterization and fluid flow distribution of the Kaimiro Formation, Taranaki Basin, New Zealand. *J. Pet. Explor. Prod. Technol.* **10**(8), 3263e–33279. 10.1007/s13202-020-01005-0 (2020).

[47] Dresser Atlas. *Log interpretation charts, Texas* 107 (Dresser industries, Inc., 1979).

[48] Poupon, A., Gaymard, R.; (1970): The evaluation of clay content from logs. 11th Ann. Soc. Prof. Well Log Analysts Logging symposium, Los Angeles (Paper SPWLA-1970-G).

[49] Asquith, G., Krygowski, D., Henderson, S., Hurley, N.; (2004): Basic Well Log Analysis (2nd edition). Vol. 16. American Association of Petroleum Geologists, Tulsa (240 pp.). 10.1306/Mth16823.

[50] Archie, G. E. The electrical resistivity log as an aid in determining some reservoir characteristics. *Transactions of the AIME* **146**(01), 54–62. 10.2118/942054-G (1942).

[51] Nabawy, B. S., Abd El Aziz, E. A., Ramadan, M. & Shehata, A. A. Implication of the micro- and lithofacies types on the quality of a gas-bearing deltaic reservoir in the Nile Delta, Egypt. *Sci. Rep.* **13**(1), 8873. 10.1038/s41598-023-35660-0 (2023). 37264046 10.1038/s41598-023-35660-0 PMC10235052

[52] Nabawy, B. S. & El Aal, A. A. Impacts of the petrophysical and diagenetic aspects on the geomechanical properties of the dolomitic sequence of Gebel El-Halal, Sinai, Egypt. *Bull. Eng. Geol. Environ.* **78**(4), 2627–2640. 10.1007/s10064-018-1264-z (2019).

[53] Safa, M. G., Nabawy, B. S., Basal, A. M. K., Omran, M. A. & Lashin, A. Implementation of a petrographical and petrophysical workflow protocol for studying the impact of heterogeneity on the rock typing and reservoir quality of reefal limestone: A case study on the nullipore carbonates in the Gulf of Suez. *Acta Geol. Sin. - Engl. Ed.* **95**(5), 1746–1762. 10.1111/1755-6724.14700 (2021).

[54] Elgendy, N. T. H., Abuamarah, B. A., Nabawy, B. S., Ghrefat, H. & Kassem, O. M. K. Pore fabric anisotropy of the Cambrian-Ordovician Nubia Sandstone in the Onshore Gulf of Suez, Egypt: A surface outcrop analog. *Nat. Resour. Res.* **29**(2), 1307–1328. 10.1007/s11053-019-09520-6 (2020).

[55] Xu, D., Zhang, S. & Qin, Y. Study of the micromechanical properties and dissolution characteristics of porous coral reef limestone. *J. Geophys. Res. Solid Earth* **129**(11), e2024JB029131 (2024).

[56] Ge, L. et al. Electromagnetic tomography for multiphase flow in the downhole annulus. *IEEE Trans. Instrum. Meas.* **74**, 1–13. 10.1109/TIM.2025.3548206 (2025).

[57] Zhang, L. et al. Permeability and stress sensitivity of coals with different fracture directions under cyclic loading–unloading conditions: A case study of the Xutuan Coal Mine in Huaibei Coalfield, China. *Rock Mech. Rock Eng.*10.1007/s00603-025-04769-1 (2025).

[58] Shenawi, S.H., White, J.P., Elrafie, E.A., Kilany, K.A.; (2007): Permeability and water saturation distribution by lithologic facies and hydraulic units: a reservoir simulation case study: society of petroleum engineers. In: 15th Society of Petroleum Engineers Middle East Oil & Gas Show and Conference, Kingdom of Bahrain. Paper no. 105273. 10.2118/105273-MS

[59] Winland H.D.; (1972): Oil accumulation in response to pore size changes, Weyburn field, Saskatchewan. Amaco Production Research Report No. F72-G-25.

[60] Gunter, G.W., Finneran, J.M., Hartmann, D.J., Miller, J.D.; (1997): January. Early determination of reservoir flow units using an integrated petrophysical method. In: SPE Annual Technical Conference and Exhibition. Society of Petroleum Engineers. 10.2118/38679-MS.

[61] Cherana, A., Aliouane, L., Doghmane, M. Z., Ouadfeul, S.-A. & Nabawy, B. S. Lithofacies discrimination of the Ordovician unconventional gas-bearing tight sandstone reservoirs using a subtractive fuzzy clustering algorithm applied on the well log data: Illizi Basin, the Algerian Sahara. *J. Afr. Earth Sci.* **196**, 104732 (2022).

[62] Dykstra, H. & Parsons, R. L. *The Prediction of Oil Recovery by Waterflooding in Secondary Recovery of Oil in the United States* 2nd edn. (API, 1950).

[63] Nabawy, B. S. An improved stratigraphic modified Lorenz (ISML) plot as a tool for describing efficiency of the hydraulic flow units (HFUs) in clastic and non-clastic reservoir sequences. *Geomech. Geophys. Geo-Energy. Geo-Resour.* **7**(3), 67. 10.1007/s40948-021-00264-3 (2021).

[64] Rahmouni, A. et al. Impacts of anisotropy coefficient and porosity on the thermal conductivity and P-wave velocity of calcarenites used as building materials of historical monuments in Morocco. *J. Rock Mech. Geotech. Eng.* **15**(7), 1687–1699. 10.1016/j.jrmge.2023.02.008 (2023).

[65] Abuhagaza, A. A., El Sawy, M. Z. & Nabawy, B. S. Integrated petrophysical and petrographical studies for characterization of reservoirs: A case study of Muglad Basin, North Sudan. *Environ. Earth Sci.* **80**(5), 171. 10.1007/s12665-021-09489-7 (2021).

[66] Safa, M.G., Omran, M.A., Basal, A.M.K., ... Kassab, M.A., Sarhan, M.A., 2025a. Amer–Al Hamd fields, central Gulf of Suez, Egypt: implications for hydrocarbon exploration. Geomechanics and Geophysics for Geo Energy and Geo Resources 11(1), 57

[67] Safa, M. G., Nabawy, B. S., Basal, A. M. K., Omran, M. A. & Kassab, M. A. Implication of the heterogeneous reservoir rock types on the reservoir quality of the nullipore reefal limestones of the Middle Miocene Belayim Formation in Al-Hamd Field, Gulf of Suez, Egypt.. *Egypt. J. Pet.* **34**(2), 149–160 (2025).

[68] Ren, Q. et al. An innovative approach to discrete facture network modeling driven by geomechanics and multiple factors.. *Geoenergy Sci. Eng.* **257**, 214200 (2026).

[69] Xue, S. et al. Visualization characterization of void structure evolution in broken limestone and its influence on permeability.. *Fuel* **405**, 136412. 10.1016/j.fuel.2025.136412 (2026).

[70] Xu, Z., Hu, X., Jiang, S., Li, X., Li, E., Zhang, W.,... Li, X. (2026). Oil-source correlation and controlling effects of oil shale on tight oil accumulation in the Triassic Chang 7 Member, Longdong Area, Ordos Basin. Petroleum Geoscience, petgeo2025-petgeo2076. 10.1144/petgeo2025-076

[71] Xu, D., Zhang, S., & Qin, Y. (2024). Study of the micromechanical properties and dissolution characteristics of porous coral reef limestone. Journal of Geophysical Research: Solid Earth, 129(11), e2024JB029131

[72] Xie, G. et al. Impact of laminae characteristics on pore-fracture connectivity in the Wufeng-Longmaxi shale. *Mar. Pet. Geol.* **182**, 107562. 10.1016/j.marpetgeo.2025.107562 (2025).

[73] Sun, M. et al. The importance of pore-fracture connectivity in overmature marine shale for methane occurrence and transportation.. *Mar. Pet. Geol.* **157**, 106495. 10.1016/j.marpetgeo.2023.106495 (2023).

[74] Li, J. et al. Mechanical properties of sandstones damaged by CO_2_ reactions and flow characteristics under complex mixed-wettability.. *Energy Fuels* **40**(4), 2108–2125 (2026).

